# Structured Immune Workup in Healthy Children With a First Episode of Severe Bacterial Infection: A 7-year Single-Center Study

**DOI:** 10.1093/infdis/jiad098

**Published:** 2023-04-11

**Authors:** Sophie Strasser, Christa Relly, Christoph Berger, Johannes Trück

**Affiliations:** Division of Infectious Diseases and Children's Research Center, University Children's Hospital Zurich, University of Zurich, Zurich, Switzerland; Division of Infectious Diseases and Children's Research Center, University Children's Hospital Zurich, University of Zurich, Zurich, Switzerland; Division of Infectious Diseases and Children's Research Center, University Children's Hospital Zurich, University of Zurich, Zurich, Switzerland; Division of Infectious Diseases and Children's Research Center, University Children's Hospital Zurich, University of Zurich, Zurich, Switzerland; Division of Immunology and Children's Research Center, University Children's Hospital Zurich, University of Zurich, Zurich, Switzerland

**Keywords:** child, immunodeficiency, meningitis, pneumonia, sepsis

## Abstract

**Background:**

Severe bacterial infections (SBIs) in otherwise healthy children are rare and may represent an underlying impairment of the immune system, including primary immunodeficiency. However, it is unclear whether and how children should be assessed.

**Methods:**

We retrospectively analyzed data from hospital records of previously healthy children aged 3 days to 18 years with SBI, including pleuropneumonia, meningitis, and/or sepsis. Patients were diagnosed or immunologically followed up between 1 January 2013 and 31 March 2020.

**Results:**

Among 432 children with SBI, findings could be analyzed in 360. Follow-up data were available for 265 children (74%), of whom 244 (92%) underwent immunological testing. Laboratory abnormalities were found in 51 of 244 patients (21%), with 3 deaths (1%). Fourteen children (6%) had immunodeficiency considered clinically relevant (3 complement deficiencies, 1 autoimmune neutropenia, 10 humoral immunodeficiencies), and 27 (11%) had milder humoral abnormalities or findings suggestive of delayed adaptive immune maturation.

**Conclusions:**

A substantial proportion of children with SBI may benefit from routine immunological testing, revealing (potentially) clinically relevant impaired immune function in 6%–17% of children. The identification of immune abnormalities allows for specific counseling of families and optimization of preventive measures, such as booster vaccinations, to avoid future SBI episodes.

## Teaser

A retrospective analysis of children with a first episode of pleuropneumonia, meningitis, or sepsis found that 6% of patients had immunodeficiency and another 11% had milder humoral abnormalities or findings suggestive of delayed adaptive immune maturation.

**(See the Editorial Commentary by Bijker and van Well on pages 1–3.)**

Severe bacterial infections (SBIs) are serious conditions and in a relevant percentage associated with underlying comorbid conditions, anatomic abnormalities, or known medical risk factors that compromise host immune function [1]. In previously healthy children, infections of such severity occur but may represent the first manifestation of a yet undiscovered transient or inborn error of immunity (IEI) [2]. Especially because the incidence of SBI with *Streptococcus pneumoniae*, *Neisseria meningitidis,* or *Haemophilus influenzae* type b (Hib) has decreased after the introduction of universal vaccination [3–7], SBI episodes—particularly in countries with high vaccination coverage, such as Switzerland [6, 7]—might rather indicate impaired immune function. Despite high sepsis-related morbidity and mortality rates in children without known comorbid conditions [8–10], little is known about the role and frequency of clinically relevant immunodeficiency, including IEI, in these patients. To our knowledge, no other study has systematically assessed children with a clinical diagnosis of SBI, independent of the causative pathogen and the clinical disease phenotype.

A previous French study examined children with Invasive pneumococcal disease (IPD) and found IEI in 10% of IPD cases [11]. The majority of patients had antibody deficiency, but 1 case of myeloid differentiation primary response protein 88 (MyD88) deficiency, 3 cases of genetically confirmed complement deficiency, and 1 case of congenital asplenia were also diagnosed. A 2022 study from the United Kingdom examining 51 patients with IPD showed similar results, with 2 cases of complement deficiency, 1 case of specific antibody deficiency, and 1 case with low levels of immunoglobulin (Ig) M, natural killer cells, and serotype-specific antipneumococcal antibodies [12]. A systematic review of various studies investigating patients with IPD demonstrated an overall IEI occurrence of 1%–10%, with rates as high as 26.4% in children >2 years of age [13]. In another approach, Picard et al followed up patients with proven MyD88 or interleukin 1 receptor–associated kinase 4 deficiency and showed that about 9 in 10 patients experienced invasive bacterial disease at least once in their lifetime, predominantly caused by pneumococci [14–17].

At the molecular level, new methods in genomics have been increasingly used to investigate children with blood culture-proven sepsis, an SBI phenotype corresponding to a high severity of infection [1, 8]. For example, fatal *Pseudomonas aeruginosa* sepsis revealed genetic variants in IEI genes in 2 of 8 patients at postmortem whole-exome sequencing [18]. Only recently, whole-exome sequencing of 172 patients with confirmed sepsis caused by *S. pneumoniae*, *S. aureus*, groups A and B *streptococcus*, *N. meningitidis,* or *H. influenzae* uncovered predicted pathogenic variants in IEI genes in 20% of cases across the entire IEI phenotype spectrum [19, 20].

However, in clinical practice, where genetic examinations are not performed routinely and pathogen isolation is often not possible (eg, owing to antibiotic pretreatment), the practical implication of these findings is limited. General guidance for the management of patients after an SBI episode is largely lacking [19, 21]. At the University Children's Hospital Zurich in Zurich, Switzerland, immunological testing of all previously healthy children presenting with proven or clinically diagnosed SBI with pleuropneumonia (PP), meningitis, and/or sepsis has been routinely performed since 2013. We evaluated data from 7 subsequent years of immunological assessment in 265 patients to characterize the role and features of immune abnormalities in otherwise healthy children after an episode of SBI.

## METHODS

### Study Design and Patient Cohort

We conducted a retrospective, single-center, cohort study using data extracted from hospital records at the University Children's Hospital of Zurich between 1 January 2013 and 31 March 2020. General consent policy was applied for further use of patient data, and data from patients whose parents or guardians had denied general consent were not further analyzed. Approval of this study was granted by the ethics committee of the Canton of Zurich (BASEC no. 2020-01065). Most children were followed up in the infectious diseases outpatient clinics in the months after SBI.

#### Inclusion Criteria

Previously healthy children aged between 3 days and 18 years who had proven or clinically diagnosed SBI with bacterial PP, meningitis, and/or sepsis were included in the study. PP was defined as severe pneumonia with signs of pleural effusion on chest radiography or sonography. The diagnosis of bacterial meningitis was made on the basis of a positive cerebrospinal fluid (CSF) culture or clinical and laboratory features consistent with bacterial meningitis [22]. We used a broad and clinical definition of sepsis, including cases of isolated bacteremia as well as children presenting with clinical signs of sepsis or septic shock in the context of a proven or suspected bacterial infection [23–25].

#### Exclusion Criteria

**Figure 1.**
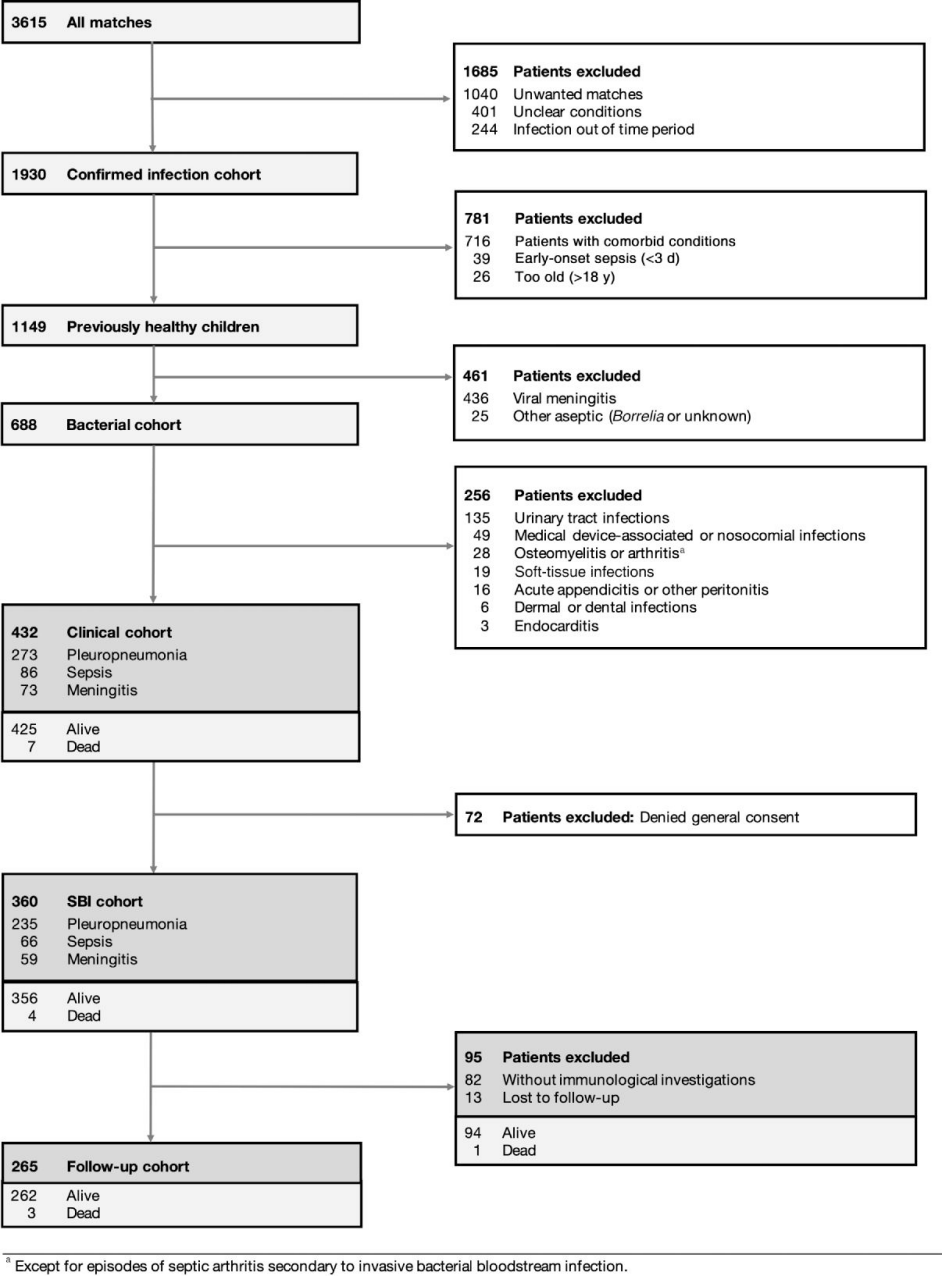
Enrollment flow diagram of severe bacterial infection (SBI) and follow-up cohorts. The SBI cohort included previously healthy children with bacterial pleuropneumonia, meningitis, and/or sepsis. For this overview, septic and nonseptic pleuropneumonia and meningitis infections were merged to single phenotypes. The follow-up cohort included patients who underwent clinical and/or laboratory immunological follow-up.

Children with SBI other than PP, meningitis, and/or sepsis were not considered, including cases of bacteremia in children with urinary tract infection or osteomyelitis, as these were not routinely assessed for IEI and outcomes would therefore be biased (Figure 1). Infections of viral, fungal, *Borrelia,* or mycobacterial origin as well as early-onset sepsis episodes (disease onset at <3 days of life) were also excluded. We specifically focused on previously healthy children and therefore excluded all episodes in children with comorbid conditions associated with susceptibility to infection or anatomic abnormalities, recent trauma, or burns.

### Data Collection and Extraction

Eligible episodes were identified by string searching in medical records during the study period for the 3 defined SBI phenotypes: bacterial PP, meningitis, and sepsis [1, 19, 26]. A complete list of search terms is available in Supplementary Table 1. A sequential filtering strategy was used, including first an automated exclusion of keywords matching the exclusion criteria (eg, cystic fibrosis or leukemia in the diagnosis list) before manual screening of the remaining data for unwanted matches (eg, aseptic), unclear conditions (eg, septic appearance without shock signs or positive culture), comorbid conditions, nonbacterial cause, and local bacterial infections. Patient history, clinical phenotype, blood count, microbiological and immunological data of remaining patients were collected, creating the clinical and SBI cohorts (Figure 1).

### Microbiology

Whenever possible, we included routinely collected relevant microbiological data alongside clinical and immunological results. The pathogens were primarily identified using cultures or bacterial PCR from blood, CSF or pleural effusion samples. Bacteria isolated from other sites (eg, sputum or stool) were only considered relevant if a clear association to the clinical condition was made and documented in the medical record.

### Immunological Testing

For the majority of patients in the follow-up cohort, immunological investigations included absolute neutrophil count and sometimes also neutrophil function (dihydrorhodamine/nitroblue tetrazolium), assessment of classic and alternative complement pathway activity, total immunoglobulin concentrations (IgG, IgA, IgM), specific antibodies against polysaccharides and proteins contained in vaccines, pocked erythrocytes (as marker for functional asplenia), and, less commonly, lymphocyte phenotyping by flow cytometry (Supplementary Table 2). Age-adjusted reference values were used to identify laboratory abnormalities, which were considered clinically significant only if samples were taken after resolution of symptoms (>1 month after diagnosis) and without any indication of ongoing inflammation to avoid false-positive findings. Abnormal complement activity was not considered relevant when a documented normal follow-up result was available.

IEI was defined as single or repetitive measurements of low IgG concentrations in patients >3 months of age, repetitive and inadequately low specific antibodies against vaccine antigens in children despite vaccination (specific antibody deficiency), genetically confirmed complement deficiency, or functionally confirmed autoimmune neutropenia [27]. Cases of repetitive low IgG measurements (with or without IgA/IgM deficiency) with documented normalization over time were classified as transient hypogammaglobulinemia of infancy. Other abnormalities, such as single measurements of neutropenia, low complement activity, low IgA or IgM, and low levels of antibodies against vaccine antibodies, which normalized or were not followed up, were classified as transient or unclear, respectively. Only patients with immunological laboratory testing were included in the analysis of frequencies of immunological abnormalities in the follow-up cohort.

## RESULTS

### Epidemiology

During the 7-year study period, we identified 432 previously healthy children with PP, meningitis, and/or sepsis. After exclusion of patients whose parents or guardians denied general consent, 360 patients with SBI were further analyzed (SBI cohort), of whom 265 (74%) underwent clinical and/or immunological follow-up (follow-up cohort). In the SBI cohort, 205 of 360 (57%) were boys. The 4 children (4 of 360 [1%]) who died were 1, 5, 6, and 23 months old. Among them were 3 patient with fatal meningitis with sepsis caused by *S. pneumoniae*, *Streptococcus agalactiae,* or *Pseudomonas aeruginosa,* and 1 with septic shock associated with a suspected bacterial superinfection in the context of varicella infection. The sepsis-related mortality rate, including only children with sepsis (n = 66), septic meningitis (n = 33), or septic PP (n = 31), was 3% (4 of 130 patients). The mortality rate for bacterial meningitis with or without bacteremia was 5% (3 of 59).

**Figure 2.**
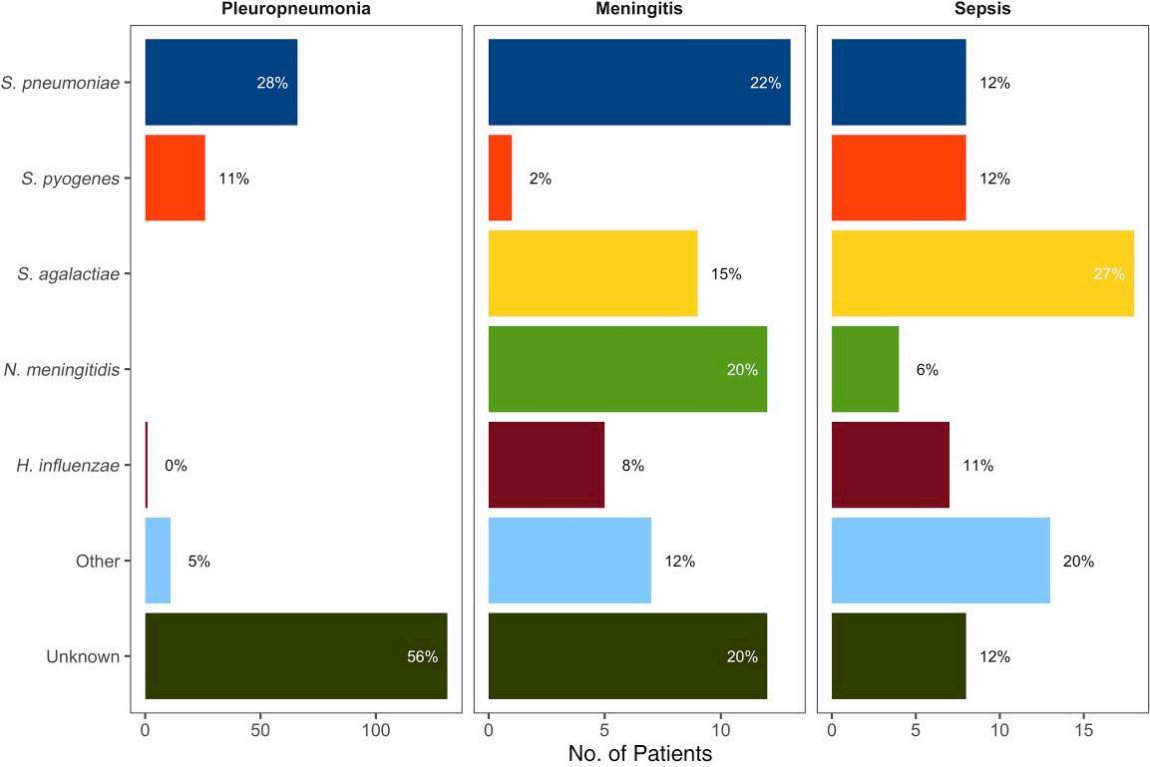
Pathogen distribution in 3 main clinical severe bacterial infection phenotypes. Cases of septic pleuropneumonia (PP) or meningitis are included in the PP and meningitis categories, respectively. One patient had combined septic PP with *Streptococcus pneumoniae* and *Haemophilus influenzae* and is therefore included in both pathogen groups. Abbreviations: *N. meningitidis, Neisseria meningitidis*; *S. agalactiae, Streptococcus agalactiae*; *S. pyogenes, Streptococcus pyogenes*.

**Figure 3.**
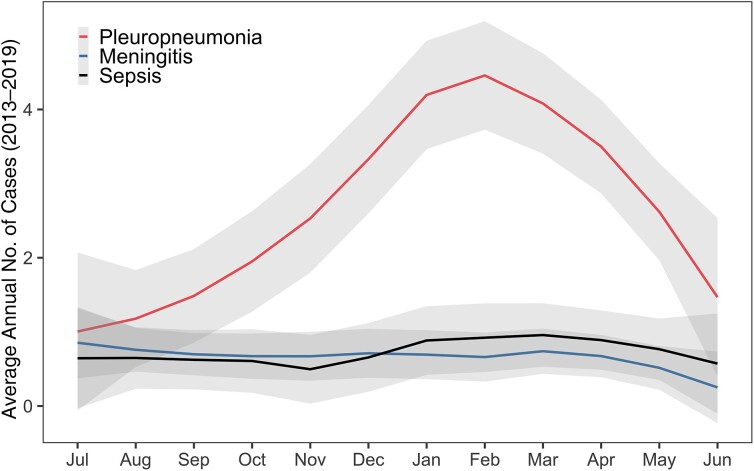
Average annual number of cases of different severe bacterial infection (SBI) phenotypes from 2013 to 2019, by month, to display seasonal variation. Standard statistics with a confidence level of 0.95 were used for graphic representation. Septic and nonseptic pleuropneumonia and meningitis infections were merged to single phenotypes. Five children with onset of infection at the end of 2012 (immunological testing in January 2013) and 25 with infection between January and March 2020 were removed from the analysis.

In 151 of 360 cases (42%), the causative pathogen remained unknown; the majority of these cases (n = 130) were nonpeptic PP (Table 1). Isolation of *S. pneumoniae* was common among all 3 SBI phenotypes, and *S. agalactiae*, *Streptococcus pyogenes, N. meningitidis* and *H. influenzae* were also frequently detected (Table 1 and Figure 2). PP infections showed a seasonal relationship, with the cumulative highest case numbers in the winter months from January to March, while no seasonality was observed for meningitis or sepsis cases (Figure 3).

**Table 1.** Clinical Characteristics and Pathogens of Severe Bacterial Infection and Follow-up Cohort

Features	SBI Cohort (n = 360)^a^		Follow-up Cohort (n = 265)^a^	
	No.	(%)	No.	(%)
Sex, male/female	205/155	(57/43)	149/116	(56/44)
Age, median (range), y	3.37	(0–15.9)	3.24	(0–15.9)
Age				
<1 y	86	(24)	64	(24)
1–4 y	153	(43)	114	(43)
5–9 y	85	(24)	63	(24)
10–17 y	36	(10)	24	(9)
SBI phenotype				
Nonseptic PP	204	(57)	146	(55)
Septic PP	31	(9)	28	(11)
Meningitis, nonseptic	26	(7)	19	(7)
Septic meningitis	33	(9)	32	(12)
Isolated sepsis	66	(18)	40	(15)
Pathogens				
Unknown	151	(42)	91	(34)
*S. pneumoniae*	87	(24)	80	(30)
*S. pyogenes*	35	(10)	29	(11)
*S. agalactiae*	27	(8)	17	(6)
*N. meningitidis*	16	(4)	14	(5)
*H. influenzae*	14	(4)	10	(4)
*M. pneumoniae*	7	(2)	4	(2)
*E. coli*	5	(1)	5	(2)
*S. aureus*	3	(1)	3	(1)
*F. necrophorum*	3	(1)	2	(1)
*S. enteritidis*	3	(1)	3	(1)
*S. typhi*	2	(1)	2	(1)
*P. aeruginosa*	2	(1)	2	(1)
Other^b^	6	(2)	4	(2)

Abbreviations: *E. coli, Escherichia coli*; *F. necrophorum*, *Fusobacterium necrophorum*; *H. influenzae, Haemophilus influenzae*; *M. pneumoniae*, *Mycoplasma pneumoniae; N. meningitidis, Neisseria meningitidis*; *P. aeruginosa, Pseudomonas aeruginosa*; PP, pleuropneumonia; *S. agalactiae, Streptococcus agalactiae*; *S. aureus, Staphylococcus aureus*; *S. enteritidis, Salmonella enteritidis*; *S. pneumoniae, Streptococcus pneumoniae*; *S. pyogenes, Streptococcus pyogenes*; *S. typhi, Salmonella typhi*; SBI, severe bacterial infection.

Data represent no. (%) of patients unless otherwise specified. One patient included in both cohorts had combined *S. pneumoniae* and *H. influenzae* infection.

Other pathogens included *Kingella kingae, Listeria monocytogenes, S. intermedius, S. mitis, Salmonella paratyphi,* and *Shigella flexneri* (1 case each).

### Microbiology

Pathogen isolation methods included bacterial culture (n = 148), polymerase chain reaction (n = 35), and pneumococcal antigen test of pleural tap (n = 25) or CSF (n = 1). Microbiological analysis was performed on blood (n = 109), pleural fluid (n = 81), CSF (n = 33), upper airway swab (n = 9) and stool (n = 2) samples. In 25 cases the pathogen was identified in both blood and CSF or pleural fluid samples. Serotype information was not available for a large proportion of pneumococcal isolates (48 of 87 [55%]). Of the samples with additional information, 13-valent pneumococcal conjugate (PCV) vaccine (PCV13) serotypes were most common (27 of 39 [69%]), with serotype 3 the most frequently identified. Of the 27 patients with SBI caused by PCV13 serotypes, 11 (41%) were fully vaccinated according to the Swiss vaccination schedule, including PCV13, while 4 (15%) were vaccinated with 7-valent PCV only and 12 (44%) had an incomplete or unknown vaccination status. Meningococcal serogroup B was most frequently detected of all grouped meningococcal isolates. For *H. influenzae*, approximately the same proportions of isolates were type b (Hib) and nontypeable (Table 2).

**Table 2.** Microbiological Details Including Serotypes/Serogroups of *Streptococcus pneumoniae*, *Neisseria meningitidis,* and *Haemophilus influenzae* in Culture-Positive Cases

Serotype/Serogroup	Pathogens (n = 210 [in 209 Patients])	
	No.	(%)
*S. pneumoniae* ^a^	87	(41)
Total serotypes^b^	88	(100)
Unknown	49	(56)
PCV13 serotypes	27	(31)
3	17	(19)
14^c^	3	(3)
19A	3	(3)
1	1	(1)
6A	1	(1)
7F	1	(1)
9V^c^	1	(1)
Non-PCV13 serotypes	12	(14)
*N. meningitidis*	16	(8)
Total serogroups	16	(100)
Unknown	3	(19)
B	8	(50)
C	1	(6)
W	3	(19)
Y	1	(6)
*H. influenzae* ^a^	14	(7)
Total serogroups	14	(100)
Unknown	1	(7)
Type b	7	(50)
Nontypeable	5	(36)
Types a, c–f	1	(7)
Other pathogens	93	(44)

Abbreviations: *H. influenzae, Haemophilus influenzae*; *N. meningitidis, Neisseria meningitidis*; PVC7, 7-valent pneumococcal conjugate vaccine (Prevenar 7); PVC13, 13-valent pneumococcal conjugate vaccine (Prevenar 13); *S. pneumoniae, Streptococcus pneumoniae*.

One patient with bacteremia had both *S. pneumoniae* (serotype 3) and *H. influenzae* (nontypeable).

One patient with bacteremia had 2 *S. pneumoniae* isolates: serotype 10B (nonvaccine) and 14 (PCV13). Nonvaccine pneumococcal serotypes included 8 (n = 1), 9N (n = 2), 10A (n = 1), 10B (n = 1), 15B or 15C (n = 2), 15C (n = 1), 24 (n = 2), 28 (n = 1), and 38 (n = 1).

Included in the PVC7 vaccine.

### Immunological Testing

In the follow-up cohort, 21of 265 patients underwent clinical assessment only, whereas 244 of 265 (92%) underwent further immunological testing. A comparison between children who were tested and those who remained untested showed no difference in sex and age but significant differences in the clinical phenotypes and pathogen distribution (Supplementary Table 3). In 3 patients laboratory testing was performed post mortem. All 3 showed laboratory abnormalities such as low immunoglobulin (IgG and/or IgM) levels or decreases in all lymphocyte subpopulations, but because the blood samples were taken during infection and genetic testing did not reveal any evidence of IEI, their clinical implication remained unclear, so these findings were not classified as significant.

In 37 other cases (15%), the results were abnormal but unclear in their clinical relevance. This included 27 patients (11%) with mild humoral abnormalities such as IgA and/or IgM deficiency (n = 21) or transient low vaccine antibody levels (n = 6) with an adequate antibody response following booster immunization. Another 10 patients (4%) showed unclear abnormalities in the innate immune system, including mildly diminished activity in classic complement pathway without follow-up testing (n = 4), transient or unclear neutropenia without follow-up testing (n = 5), and elevated pocked erythrocytes without further testing to assess (functional) asplenia (n = 1).

Immunological laboratory abnormalities indicative of IEI were found in 14 patients (6%), and to a greater extent in patients with sepsis or septic meningitis and in those aged <1 year compared with the entire follow-up cohort (Table 3). Most of these 14 children (9 of 239 [4%]) had hypogammaglobulinemia, including 4 cases of transient hypogammaglobulinemia of infancy in 3 girls and 1 boy <6 months of age with late-onset *S. agalactiae* sepsis. The 3 girls showed other signs of immunological immaturity, such as transient low anti-Hib antibody levels despite regular vaccination according to the Swiss immunization schedule, which resolved after booster vaccination, or associated transient (presumed autoimmune) neutropenia. Specific antibody deficiency was diagnosed in 1 patient with Hib sepsis, with low levels of anti-Hib antibodies despite repeated vaccination.

**Table 3.** Immunological Abnormalities in the Follow-up Cohort Stratified by Clinical Phenotype

ID Group or Outcome^a^	Follow-up Cohort (n = 265)		Clinical Phenotype										Age, y								Sex			
			PP (n = 146)		Septic PP^b^ (n = 28)		Meningitis (n = 19)		Septic Meningitis^b^ (n = 32)		Isolated Sepsis (n = 40)		<1 (n = 64)		1–4 (n = 114)		5–9 (n = 63)		10–17 (n = 24)		Male (n = 149)		Female (n = 116)	
No laboratory testing, no.	21	…	16	…	0	…	2	…	0	…	3	…	1	…	6	…	7	…	7	…	14	…	7	…
Laboratory testing, no. (%)	244	(100)	130	(100)	28	(100)	17	(100)	32	(100)	37	(100)	63	(100)	108	(100)	56	(100)	17	(100)	135	(100)	109	(100)
No laboratory abnormalities	190	(78)	115	(79)	22	(79)	14	(82)	18	(56)	21	(57)	37	(59)	88	(81)	51	(91)	14	(82)	102	(76)	88	(81)
Any abnormality	54	(22)	15	(10)	6	(21)	3	(18)	14	(44)	16	(43)	26	(41)	20	(19)	5	(9)	3	(18)	33	(24)	21	(19)
Definite IEI	14	(6)	2	(1)	1	(4)	0	(0)	**6** ^c^	**(19)**	**5**	**(14)**	**10**	**(16)**	1	(1)	1	(2)	2	(12)	9	(7)	5	(5)
Unclear/transient ID: humoral	27	(11)	7	(5)	4	(14)	2	(12)	**7**	**(22)**	**7**	**(19)**	**12**	**(19)**	11	(10)	3	(5)	1	(6)	14	(10)	13	(12)
Unclear/transient ID: innate	10	(4)	6	(4)	1	(4)	1	(6)	0	(0)	2	(5)	2	(3)	7	(6)	1	(2)	0	(0)	8	(6)	2	(2)
Death^d^	3	(1)	0	(0)	0	(0)	0	(0)	1	(3)	2	(5)	2	(3)	1	(1)	0	(0)	0	(0)	2	(1)	1	(1)

Abbreviations: ID, immunodeficiency; PP, pleuropneumonia.

Children without laboratory testing were clinically assessed by an immunology/infectious diseases specialist. Definite inborn errors of immunity (IEIs) included genetically confirmed complement deficiency, autoimmune neutropenia, and predominantly antibody disorders.

Defined as PP or meningitis with a positive blood culture and/or clinically characterized as “septic shock.”

Boldface values signify clinical and demographic categories in which patients with relevant immunodeficiencies or humoral abnormalities were overrepresented.

Postmortem laboratory testing showed low lymphocyte counts and/or low immunoglobulin levels, but these were not categorized as abnormal owing to ongoing infection; postmortem genetic investigations did not reveal definite IEI diagnoses.

Complement deficiencies included a C2 deficiency, a C7 deficiency, and a properdin deficiency, which were genetically confirmed after 2 consecutive measurements of low complement activity. One case of autoimmune neutropenia with detection of FcγRIIIb antibodies was reported in a girl after *S. pneumoniae* sepsis (Table 4). There was no obvious overrepresentation of vaccine failure cases in patients with immunological abnormalities, although numbers of vaccine failures were low and a formal analysis not possible (Supplementary Table 4).

**Table 4.** Type of Immunological Abnormality by Test Category

Abnormality or Outcome	No. Tested	No. With Abnormality (any) or Outcome	No. With IEI^a^	Details
**Any abnormality**	**244**	**54**	**14**	
**Death**		**3**	**0**	
**Innate immunity**	**244**	**14**	**4**	
Low complement activity	228	7	3	
Reduced activity in classic pathway		4	0	Low classic complement activity without follow-up visit
C2 deficiency		1	1	In a 1-mo-old boy after *S. agalactiae* sepsis and meningitis
C7 deficiency		1	1	In a 13-y-old boy after *N. meningitidis* (B) sepsis and meningitis
Properdin deficiency		1	1	In a 9-mo-old boy after *S. pneumoniae* PP
Low ANC	233	6	1	…
Transient neutropenia		4	0	Measurement of low ANC ≥1 mo after infection with normalization in follow-up visit
Unclear neutropenia		1	0	Measurement of low ANC ≥1 mo after infection without follow-up visit
Autoimmune neutropenia		1	1	In a 12-mo-old girl after *S. pneumoniae* sepsis
Unclear increased pocked erythrocytes	213	1	0	High percentage of pocked erythrocytes (5.9%) after *S. pneumoniae* PP without follow-up visit
Neutrophil dysfunction (CGD)	22	0	0	
**Antibody deficiency**	**244**	**37**	**10**	
Hypogammaglobulinemia	239^b^	9	9	
Transient hypogammaglobulinemia of infancy		4	4	In 3 patients aged >3 mo after *S. agalactiae* sepsis, with transient neutropenia (n = 1), with low anti-PS antibodies (n = 2)
IgG deficiency with or without low IgA or IgM		5	5	After Hib sepsis (n = 1), with low IgA after GBS/*S. pneumoniae* sepsis (n = 2), with low IgM after GBS sepsis/PP with unknown pathogen (n = 2)
Selective Ig deficiencies	226^c^	21	0	
Selective IgA deficiency		10	0	In patients aged ≥3 mo with low age-adjusted IgA levels, with unclear reduced activity in classic pathway (n = 2)
Selective IgM deficiency		8	0	In patients aged ≥3 mo with low age-adjusted IgM levels
Low IgA and IgM		3	0	Patients aged ≥3 mo with low age-adjusted IgA and IgM levels
Low anti-polysaccharide antibodies	218	6	1	
Specific antibody deficiency		1	1	In a 4-y-old boy after Hib sepsis with low anti-Hib antibodies despite repeated vaccinations
Transient low anti-PS IgG		3	0	Transient low anti-Hib antibodies despite vaccination but adequate rise after booster vaccination, with transient neutropenia (n = 1)
* *Transient low anti-PS IgG with low IgA/IgM		2	0	In patients aged ≥3 mo with low age-adjusted IgA and IgM levels and transient low anti-Hib antibodies
Low antiprotein antibodies	230	1	0	Transient low antitetanus IgG despite vaccination but adequate rise after booster vaccination
**T-cell deficiency**	**31**	**0**	**0**	

Abbreviations: ANC, absolute neutrophil count; CGD, chronic granulomatous disease; GBS, group B *Streptococcus* (*S. agalactiae)*; Hib, *Haemophilus influenzae* type b; Ig, immunoglobulin, *N. meningitidis, Neisseria meningitidis*; IEI, inborn error of immunity; PP, pleuropneumonia; PS, polysaccharide; *S. agalactiae, Streptococcus agalactiae*; *S. pneumoniae, Streptococcus pneumoniae*.

Definite IEI per the latest International Union of Immunological Societies definitions and/or clinically relevant findings consistent with impaired immune function.

Total IgG tested.

Total IgA/IgM tested.

## DISCUSSION

This retrospective analysis of 7 years of routine immunological testing in previously healthy children with bacterial PP, meningitis, and/or sepsis, laboratory investigations during follow-up revealed significantly impaired immune function in 6% of cases and potentially clinical relevant milder humoral abnormalities or findings suggestive of delayed adaptive immune maturation in a further 11% [20, 28].

The findings in this work strengthen the approach to routinely investigate previously healthy children with SBIs such as sepsis or meningitis. Most laboratory abnormalities that were detected in our study affected the humoral immune system of young children and were at least partially transient, suggesting a maturation disorder of the B-cell system that has no known genetic origin in the majority of cases [20]. Our results are consistent with previous work [11, 13] and underline the importance of immunological risk factors in the pathophysiology of invasive bacterial infections in seemingly healthy children [2, 29–31] and the need for a standardized approach when investigating these children for immune disorders.

The overall mortality rate in our cohort was low (1%), as the majority of patients presented with nonseptic PP, for which fatal outcomes are rare [26]. However, the fatality rate for sepsis alone (isolated or septic PP or meningitis) was 3 times higher (3%), which is similar to the findings in the Swiss Pediatric Sepsis Study, a national study including all children with bacteremia between 2012 and 2015 [1, 19].

Four children (2%) in our cohort had an innate immunity disorder diagnosed, similar to the rate in a previous study that found 5 children (3%) with innate immunity disorder among patients with IPD [11]. However, in our study we did not test interleukin 6 production by white blood cells, potentially missing out on MyD88 deficiency and other defects of Toll-like receptor signaling [14, 16, 17].

The proportion of patients with immunological abnormalities varied with the clinical phenotype. Children who experienced an episode with sepsis or septic meningitis had a 2-fold higher chance of having abnormal laboratory investigations or being diagnosed with an immunodeficiency. Similarly, those <1 year old had a 2-fold higher risk of an IEI diagnosis than older children. However, because sepsis and septic meningitis were more common in very young children, the separate effects of age and clinical phenotypes cannot be clearly distinguished (Supplementary Figure 1).

On the other hand, children with PP episodes without sepsis showed the lowest rates of relevant immunological abnormalities (1%). In this same group, microbiological information was lowest, potentially as a result of prior antimicrobial treatment, but it cannot be excluded that the infection was nonbacterial, for which typical preexisting immunological abnormalities, such as antibody production disorders, may be less relevant. Children with a single PP episode and (minor) effusion who do not undergo therapeutic or diagnostic drainage may be less likely to have relevant results from routine immunological testing, so a separate cost-benefit assessment is required for this population. On the other hand, because children with PP episodes are usually older than those with sepsis or meningitis, immunological abnormalities are less likely to be due to delayed immune maturation and therefore more likely to be persistent.

In accordance with prior work [11, 12], isolated IgA and/or IgM deficiency was common in our study. Although the clinical relevance of these findings remains unclear, both our study and that of Gaschignard et al [11] demonstrated an unusually high rate of IgA deficiency in children who had an SBI (5%–7%) compared with the general population (<1%) [32]. Similarly, a 2021 study showed a higher rate of IgA deficiency in children with recurrent respiratory tract infections compared with healthy controls [33], while at least some patients with selective IgM deficiency may go on to be diagnosed with an IEI [34].

The recognition of even minor immunological abnormalities can be important, and we therefore suggest routinely performing immunological investigations in children after recovery (eg, after 1–2 months) following an episode of invasive bacterial infection, such as PP, meningitis, or sepsis. Children with complement deficiencies or antibody production disorders may benefit from additional vaccine doses, supplementary vaccines, or antibiotic prophylaxis for at-risk populations as well as alerting of parents and primary care physicians and establishment of emergency plans.

Genetic testing was not routinely performed in our patients because this was not part of routine care. A more sensitive approach may be to combine routine immunological investigations with broad genetic testing. Previous work using high-throughput sequencing methods has shown that monogenic conditions may be more common than previously thought [19, 29–31]. Nevertheless, functional confirmation of genetic variants in immune genes remains important and challenging. In addition, genetic testing may also miss a large proportion of children with nongenetic delayed immune maturation, as was most seen in the present study.

This study has several limitations. First, its retrospective nature is subject to bias and, despite a systematic follow-up approach, not all children underwent the same investigations. We attempted to minimize selection bias by deliberately choosing a broad filter strategy but cannot exclude the possibility that children with more severe infections were more likely to be investigated. Second, clinical phenotypes were based on information available in our clinical information system. Particularly the assessment of pleuraleffusion in children with pneumonia, thereby defining it as PP rather than pneumonia, may not be consistent. In addition, we used a broad definition of sepsis as used by the clinical team managing the child rather than one based on strict criteria, such as the presence of organ dysfunction [24, 25]. On the other hand, the use of clinical definitions may be more useful, and our results may be overall more applicable in a clinical setting.

In conclusion, this study on routine immunological investigations after a first episode of SBI in previously healthy children revealed significant impaired immune function in 6% of cases and potentially clinically relevant humoral immune abnormalities in a further 11%. Most of these children had sepsis and/or meningitis, while fewer children with PP had abnormal immunological results. The results of our study suggest that screening all children for immunological abnormalities after SBI, especially in children with bacterial meningitis and sepsis, seems useful as it allows for targeted counseling of families and optimization of preventive measures, such as booster vaccinations, to avoid future SBI episodes.

## Supplementary Data

Supplementary materials are available at *The Journal of Infectious Diseases* online. Consisting of data provided by the authors to benefit the reader, the posted materials are not copyedited and are the sole responsibility of the authors, so questions or comments should be addressed to the corresponding author.

## Supplementary Material

jiad098_Supplementary_Data

## Notes

***Author contributions*.** Study concept and design: S. S., C. B., and J. T. Definition of the diagnostic algorithm: C. R. and C. B. Acquisition and analysis of data: S. S. and J. T. Interpretation of data: S. S., C. B, and J. T. Drafting of manuscript: S. S. and J. T. Revision of manuscript: all authors.
