# Supplementary material for: Structured Immune Workup in Healthy Children With a First Episode of Severe Bacterial Infection: A 7-year Single-Center Study
Source: J Infect Dis. 2023 Apr 11;228(1):8–17. doi: 10.1093/infdis/jiad098 (PMC10304764; doi:10.1093/infdis/jiad098)
Supplement: jiad098_Supplementary_Data [file jiad098_supplementary_data.docx]

**Supplementary material**

**Supplementary table 1.** German SBI search terms for phenotypes bacterial pleuropneumonia, meningitis and sepsis.

| Phenotype | Search terms |
| --- | --- |
| Pleuropneumonia | Schwere Pneumonie, Pleuropneumonie, Pleurapneumonie, Parapneumonisch, Pleuraempyem |
| Sepsis | Sepsis, Septischer Schock, Septisch, Septikämie, Bakteriämie, SIRS |
| Meningitis | Meningitis, Hirnhautentzündung |

**Supplementary table 2.** Routine investigations for healthy children with a first episode of severe bacterial infection.

| **Routine investigations** |
| --- |
| Whole blood count with differential (including absolute neutrophil count) |
| Pocked erythrocytes |
| Classical and alternative complement pathway activity |
| Immunoglobulin concentrations (IgA/IgM/IgG) |
| Specific IgG antibody concentrations against vaccine antigens  - polysaccharides, usually *H. influenzae* type B, pneumococcal serotypes  - proteins, usually tetanus toxin |

**Supplementary table 3.** Distribution of clinical characteristics and pathogens in tested (follow-up cohort) and untested children with SBI.

| Features | Not tested  (n = 95) | | Follow-up cohort (n = 265) | | p-value |
| --- | --- | --- | --- | --- | --- |
|  |  |  |  |  |  |
| **Male, n (%)** | 56 | (59) | 149 | (56) | **0.74** |
| **Age, n (%)** |  |  |  |  | **0.80** |
| < 1 year | 22 | (23) | 64 | (24) |  |
| 1-4 years | 39 | (41) | 114 | (43) |  |
| 5-9 years | 22 | (23) | 63 | (24) |  |
| 10-17 years | 12 | (13) | 24 | (9) |  |
| **SBI phenotype, n (%)** |  |  |  |  | **p<0.001** |
| PP, non-septic | 58 | (61) | 146 | (55) |  |
| Septic PP | 3 | (3) | 28 | (11) |  |
| Meningitis, non-septic | 7 | (7) | 19 | (7) |  |
| Septic meningitis | 1 | (1) | 32 | (12) |  |
| Isolated sepsis | 26 | (27) | 40 | (15) |  |
| **Pathogens, n (%)** |  |  |  |  | **p<0.001** |
| Unknown | 60 | (63) | 91 | (34) |  |
| *S. pneumoniae* | 7 | (7) | 80 | (30) |  |
| *S. pyogenes* | 6 | (6) | 29 | (11) |  |
| *S. agalactiae* | 10 | (11) | 17 | (6) |  |
| *N. meningitidis* | 2 | (2) | 14 | (5) |  |
| *H. influenzae* | 4 | (4) | 10 | (4) |  |
| Other | 6 | (6) | 25 | (9) |  |

Note: Standard statistical analysis (chi-square test for distribution) was used for the calculation of p-values. One patient with combined *S. pneumoniae* and *H. influenzae* infection in PID cohort (included in both pathogen groups). Abbreviations: SBI, severe bacterial infection

**Supplementary table 4.** Immunological abnormalities in children with SBI (n=360) with unknown pathogen/serotype/serogroup, non-vaccine preventable pathogen or vaccine preventable pathogen with prior adequate vaccination (= vaccine failure).

| ID group, n (%) | Unknown pathogen/serotype (n = 204) | | Non-vaccine preventable pathogens^1^ (n = 144) | | Vaccine failure (n = 12) | |  |
| --- | --- | --- | --- | --- | --- | --- | --- |
|  |  |  |  |  |  |  |  |
| Not tested | 78 | (38) | 38 | (26) | 0 | (0) |  |
| No abnormalities | 107 | (52) | 75 | (52) | 8 | (67) |  |
| Relevant ID | 3 | (1) | 10 | (7) | 1 | (8) |  |
| Unclear/transient ID | 14 | (7) | 20 | (14) | 3 | (25) |  |
| Death | 2 | (1) | 1 | (1) | 0 | (0) |  |
| ^1^ Includes non-vaccine pathogens/serotypes as well as unvaccinated or incompletely vaccinated children. | | | | | | |  |

Note: Vaccine failure was defined for children ≤ 5 years of age who had an SBI with a vaccine-preventable serotype of *S. pneumoniae* or serogroup of *N. meningitidis*/*H. influenzae* in children who had been fully vaccinated according to the Swiss national immunization schedule.

Abbreviations: SBI, severe bacterial infection; ID, immunodeficiency.


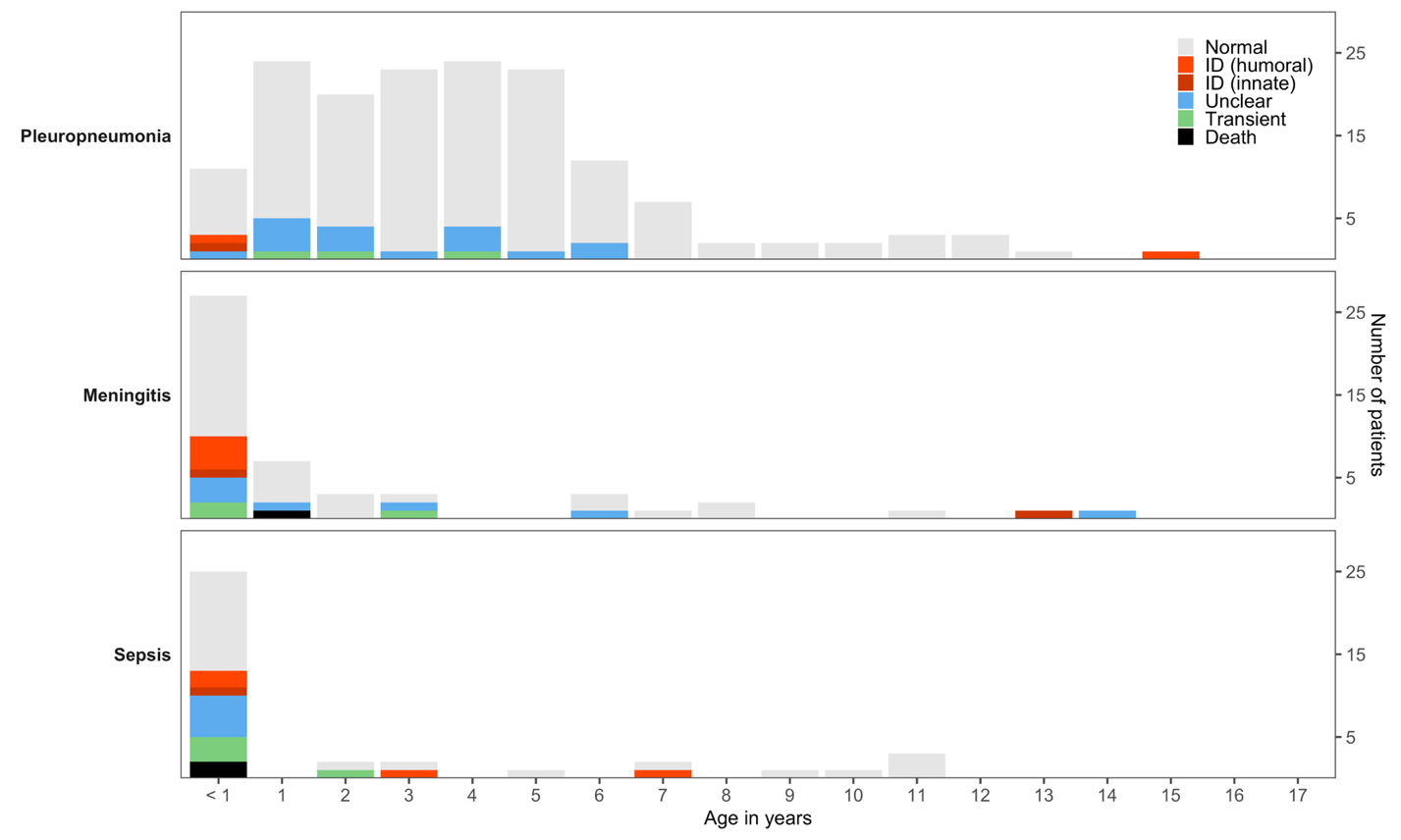


**Supplementary figure 1.** Age distribution and immunological results of children with immunological testing (n=244). Cases of septic pleuropneumonia/meningitis are only included in the category pleuropneumonia and meningitis, respectively. Cases of transient hypogammaglobulinemia of infancy are included in the category relevant ID (humoral). Abbreviations: ID, immunodeficiency.
